# Applications of Three Dithienylpyrroles-Based Electrochromic Polymers in High-Contrast Electrochromic Devices

**DOI:** 10.3390/polym9030114

**Published:** 2017-03-22

**Authors:** Yuh-Shan Su, Jui-Cheng Chang, Tzi-Yi Wu

**Affiliations:** 1 Department of Chemical and Materials Engineering, National Yunlin University of Science and Technology, Yunlin 64002, Taiwan; d10115003@yuntech.edu.tw; 2 Department of Chemical Engineering, National Cheng Kung University, Tainan 70101, Taiwan; d700215@gmail.com

**Keywords:** electrochemical polymerization, optical contrast, spectroelectrochemistry, coloration efficiency, electrochromic devices

## Abstract

Three dithienylpyrroles (1-(4-(methylthio)phenyl)-2,5-di(thiophen-2-yl)-pyrrole (MPS), 1-(4-methoxyphenyl)-2,5-di(thiophen-2-yl)-pyrrole (MPO), and 4-(2,5-di(thiophen-2-yl)-pyrrol-1-yl)benzonitrile (ANIL)) were synthesized and their corresponding polydithienylpyrroles (PSNS) were electrosynthesized using electrochemical polymerization. Spectroelectrochemical studies indicated that poly(1-(4-(methylthio)phenyl)-2,5-di(thiophen-2-yl)-pyrrole) (PMPS) film was green, dark green, and brown in the neutral, oxidation, and highly oxidized state, respectively. The incorporation of a MPS unit into the PSNS backbone gave rise to a darker color than those of the MPO and ANIL units in the highly oxidized state. The PMPS film showed higher Δ*T*_max_ (54.47% at 940 nm) than those of the PMPO (43.87% at 890 nm) and PANIL (44.63% at 950 nm) films in an ionic liquid solution. Electrochromic devices (ECDs) employing PMPS, PMPO, and PANIL as anodic layers and poly(3,4-(2,2-diethypropylenedioxy)thiophene)(PProDOT-Et_2_) as a cathodic layer were constructed. PMPO/PProDOT-Et_2_ ECD showed the highest Δ*T*_max_ (41.13%) and coloration efficiency (674.67 cm^2^·C^−1^) at 626 nm, whereas PMPS/PProDOT-Et_2_ ECD displayed satisfactory Δ*T*_max_ (32.51%) and coloration efficiency (637.25 cm^2^·C^−1^) at 590 nm. Repeated cyclic voltammograms of PMPS/PProDOT-Et_2_, PMPO/PProDOT-Et_2_, and PANIL/PProDOT-Et_2_ ECDs indicated that ECDs had satisfactory redox stability.

## 1. Introduction

π-conjugated polymers have drawn great attention from researchers in recent years due to their wide use in academic and industrial applications, such as electrochromic devices (ECD) [1,2,3], thin-film polymer solar cells [4], sensing materials [5,6], polymeric memory devices [7,8], catalysts for methanol and ethanol oxidation reactions [9,10,11], polymeric light-emitting diodes [12,13], and smart windows [14]. Among them, the benefit of ECDs is their very low power consumption. Moreover, the redox state of ECDs exists with almost no input of power upon changing color, which is called a “memory effect”. Two types of electrochromic materials are currently used in ECDs: inorganic electrochromic materials (transition metal oxides) and organic electrochromic materials (viologens, conducting π-conjugated polymers, metallopolymers, and metallophthalocyanines) [15]. Compared to transition metal oxides, π-conjugated polymers display satisfactory long-term stability, high optical contrast, high coloration efficiency, and a wide range of colors. In the past decade, the most commonly studied π-conjugated polymers have been polypyrroles [16], polythiophenes [17,18], polyanilines [19], and polycarbazoles [20,21]. In recent years, Toppare and Cihaner et al. reported a series of dithienylpyrrole (SNS) derivatives and investigated their electro-optical properties and electrochromic behaviors [22,23]. The incorporation of a pyrrole ring between two thiophene units increases the electron donating ability of the polymer backbone and decreases the onset potential of polymer films.

Up to now, the incorporation of a 4-(methylthio)aniline unit into a poly(dithienylpyrrole) backbone and the comparison of its effects with alkoxy-phenyl and cyano-phenyl substituents on the electrochromic, spectroelectrochemical, and ECD properties has not been reported. The purpose of this paper is to synthesize a thiomethylphenyl-based anodic polymer (PMPS) via electrochemical polymerizations and compare its spectroelectrochemical properties, coloration efficiency, electrochromic switching, and colorimetry with PMPO and PANIL. Moreover, the benefits of ionic liquids (ILs) such as non-volatility, high conductivity, and a wide potential window make them easy alternatives as stable electrolytes in electrochemical devices [24,25,26,27,28]. In this paper, ECDs were prepared using PMPS, PMPO, and PANIL as the electrochromic materials of anodic electrodes, PProDOT-Et_2_ as the electrochromic material of the cathodic electrode, and an ionic liquid/polymer composite membrane as the electrochromic electrolyte. The spectroelectrochemistry, electrochromic switching, colorimetry, coloration efficiency, open circuit memory, and redox stability of PMPS/PProDOT-Et_2_, PMPO/PProDOT-Et_2_, and PANIL/PProDOT-Et_2_ ECDs were studied in detail.

## 2. Materials and Methods 

### 2.1. Materials and Electrochemical Synthesis

All chemicals and reagents in this paper were purchased from Acros (Morris Plains, NJ, USA), TCI (Tokyo, Japan), and Sigma-Aldrich (St. Louis, MO, USA), and were used as received. 3,3-diethyl-3,4-dihydro-2H-thieno [3,4–b][1,4]dioxepine (ProDOT-Et_2_), 1-ethyl-3-propylimidazolium bis(trifluoromethanesulfonyl)imide ([EPI^+^][TFSI^−^]), and 1,4-di(2-thienyl)-1,4-butanedione were synthesized from previously published procedures [29,30,31]. PMPS, PMPO, and PANIL films were prepared potentiostatically at 0.9 V on Indium Tin Oxide (ITO) glass electrodes with a charge density of 20 mC·cm^−2^. PVdF-HFP/ionic liquid composite electrolytes were prepared according to previously published work [32].

#### 2.1.1. Synthesis of 1-(4-(Methylthio)phenyl)-2,5-di(thiophen-2-yl)-pyrrole (MPS)

**Figure 1:**
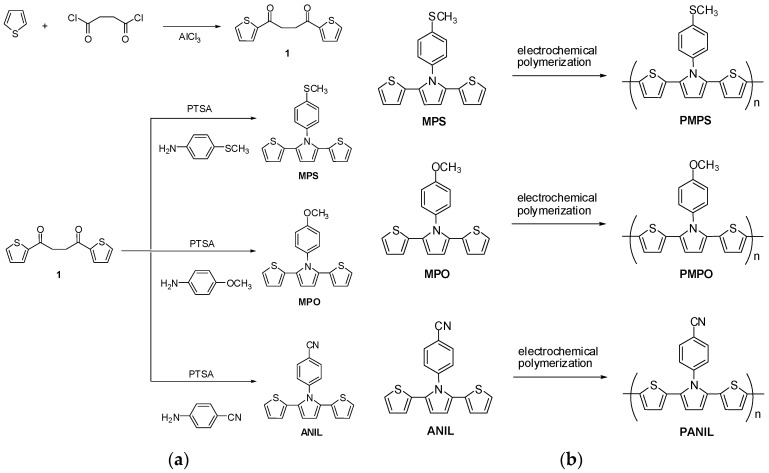
(**a**) The synthetic routes of poly(2,5-dithienylpyrrole) derivatives; (**b**) The electrochemical polymerization of poly(2,5-dithienylpyrrole) derivatives.

1,4-di(2-thienyl)-1,4-butanedione (1.25 g, 5 mmol), 4-(methylthio)aniline (1.06 g, 7 mmol), *p*-toluenesulfonic acid (0.1 g, 0.58 mmol), and 25 mL toluene were added in a round bottom flask and stirred at 110 °C under Argon for 24 h. After cooling, toluene was evaporated and the crude product was purified using column chromatography (silica gel, dichloromethane: hexane = 1:1) to give the desired MPS. Yield: 61%. ^1^H NMR (700 MHz, DMSO-*d*_6_): δ7.34 (d, *J* = 8.7 Hz, 2H, phenyl-H), 7.31 (dd, *J* = 5.2 and 1.4 Hz, 2H, Th-H), 7.28 (d, *J* = 8.7 Hz, 2H, phenyl-H), 6.90 (dd, *J* = 5.2 and 3.7 Hz, 2H, Th-H), 6.71 (dd, *J* = 3.7 and 1.4 Hz, 2H, Th-H), 6.56–6.57 (m, 2H, Py-H), 2.52 (s, 3H, –SCH_3_). Elemental analysis: Calculated (Elem. Anal. Calcd.) for C_19_H_15_NS_3_: C, 64.55%; H, 4.28%; N, 3.96%. Found: C, 64.35%; H, 4.22%; N, 3.85%. The synthetic routes of MPS are shown in Figure 1.

#### 2.1.2. Synthesis of 1-(4-Methoxyphenyl)-2,5-di(thiophen-2-yl)-pyrrole (MPO)

MPO was synthesized using a similar procedure to that of MPS. Yield: 65%. ^1^H NMR (700 MHz, DMSO-*d*_6_): δ7.28 (dd, *J* = 3.7 and 1.4 Hz, 2H, Th-H), 7.27 (d, *J* = 8.5 Hz, 2H, phenyl-H), 7.04 (d, *J* = 8.5 Hz, 2H, phenyl-H), 6.88 (dd, *J* = 5.1 and 3.7 Hz, 2H, Th-H), 6.71 (dd, *J* = 3.7 and 1.4 Hz, 2H, Th-H), 6.55–6.56 (m, 2H, Py-H), 3.83 (s, 3H, –OCH_3_). Elem. Anal. Calcd. for C_19_H_15_NOS_2_: C, 67.62%; H, 4.48%; N, 4.15%. Found: C, 67.55%; H, 4.39%; N, 4.06%.

#### 2.1.3. Synthesis of 4-(2,5-Di(thiophen-2-yl)-pyrrol-1-yl)benzonitrile (ANIL)

ANIL was synthesized using a similar procedure to that of MPS and MPO. Yield: 58%. ^1^H NMR (700 MHz, DMSO-*d*_6_): δ7.98 (d, *J* = 8.4 Hz, 2H, phenyl-H), 7.57 (d, *J* = 8.4 Hz, 2H, phenyl-H), 7.36 (dd, *J* = 5.2 and 1.2 Hz, 2H, Th-H), 6.91 (dd, *J* = 5.2 and 3.4 Hz, 2H, Th-H), 6.67(dd, *J* = 3.4 and 1.2 Hz, 2H, Th-H), 6.59–6.60 (m, 2H, Py-H). Elem. Anal. Calcd. for C_19_H_12_N_2_S_2_: C, 68.64%; H, 3.64%; N, 8.43%. Found: C, 68.55%; H, 3.68%; N, 8.27%.

### 2.2. Construction of ECDs and Spectroelectrochemical Characterizations

The electrochemical and spectroelectrochemical properties of PMPS, PMPO, and PANIL films coated on the working electrodes and PMPS/PProDOT-Et_2_, PMPO/PProDOT-Et_2_, and PANIL/PProDOT-Et_2_ ECDs were investigated using a CHI660a electrochemical analyzer (CH Instruments, Austin, TX, USA) and a V-630 JASCO UV-Visible spectrophotometer (JASCO International Co., Ltd., Tokyo, Japan).

ECDs were built using PMPS, PMPO, or PANIL as the anodically coloring material, PProDOT-Et_2_ as the cathodically coloring material, and PVdF-HFP/ionic liquid composite membranes as electrolytes. PMPS, PMPO, and PANIL films were electrodeposited potentiostatically onto ITO-coated glasses at +0.9 V, and PProDOT-Et_2_ were electrodeposited onto ITO-coated glasses at +1.4 V. Film thicknesses of the deposited anodic and cathodic layers were obtained with an Alpha-Step profilometer (KLA Tencor D-120, KLA-Tencor, Milpitas, CA, USA). The approximate average thicknesses of anodic and cathodic layers are 100–105 nm. ECDs were assembled by anodic and cathodic polymers facing each other and were separated by PVdF-HFP/ionic liquid composite membranes.

## 3. Results and Discussion

### 3.1. Electrochemical Polymerizations of Anodic Polymer Films

**Figure 2:**
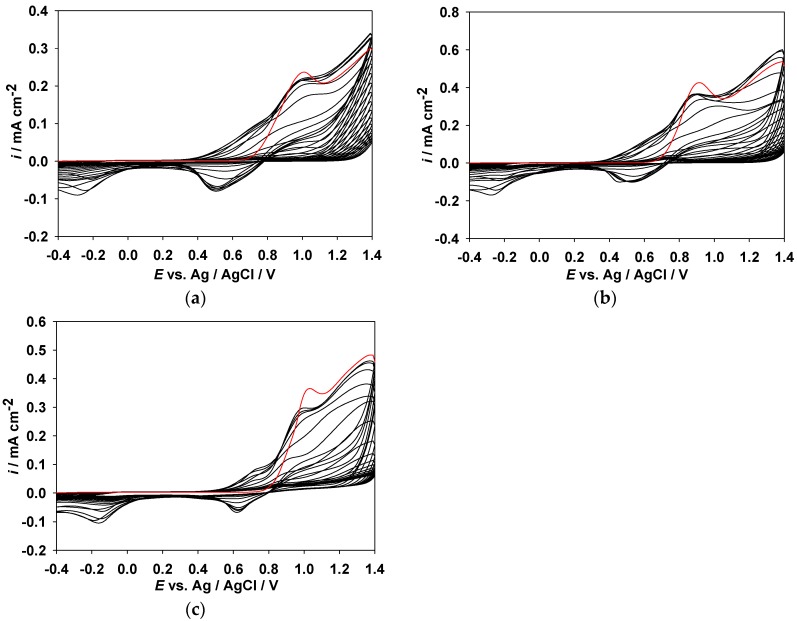
Cyclic voltammograms of 2 mM (**a**) MPS; (**b**) MPO, and (**c**) ANIL in 0.1 M LiClO_4_/EtOH/EA at a scan rate of 100 mV·s^−1^ on an ITO working electrode. The red line indicates the first cycle of CVs.

The cyclic voltammogram (CV) curves of MPS, MPO, and ANIL in EtOH/EA (1:1, by volume) solution containing 0.1 M LiClO_4_ are shown in Figure 2, after scanning the potentials between −0.4 and 1.4 V at a scan rate of 100 mV·s^−1^ continuously for 20 cycles. PMPS, PMPO, and PANIL were electrodeposited onto the surface of the ITO working electrode, and the synthetic routes of PMPS, PMPO, and PANIL are displayed in Figure 1. The onset potentials of MPS, MPO, and ANIL are 0.7, 0.69, and 0.81 V, respectively. The onset potential of MPS is comparable to MPO, implying the incorporation of the methylthio-phenyl unit on the nitrogen atom of the pyrrole ring that shows a similar electron donating property to that of the methoxyphenyl unit. However, the incorporation of the benzonitrile unit on the nitrogen atom of the pyrrole ring shows a larger onset potential than those of the methylthio-phenyl and methoxyphenyl units, implying the incorporation of an electron withdrawing benzonitrile unit that increases the onset potential significantly. The oxidation peaks of PMPS, PMPO, and PANIL are located at 0.95, 0.9, and 1.0 V, respectively, whereas the reduction peaks of PMPS, PMPO, and PANIL appear at 0.5, 0.55, and 0.6 V, respectively. 

**Figure 3:**
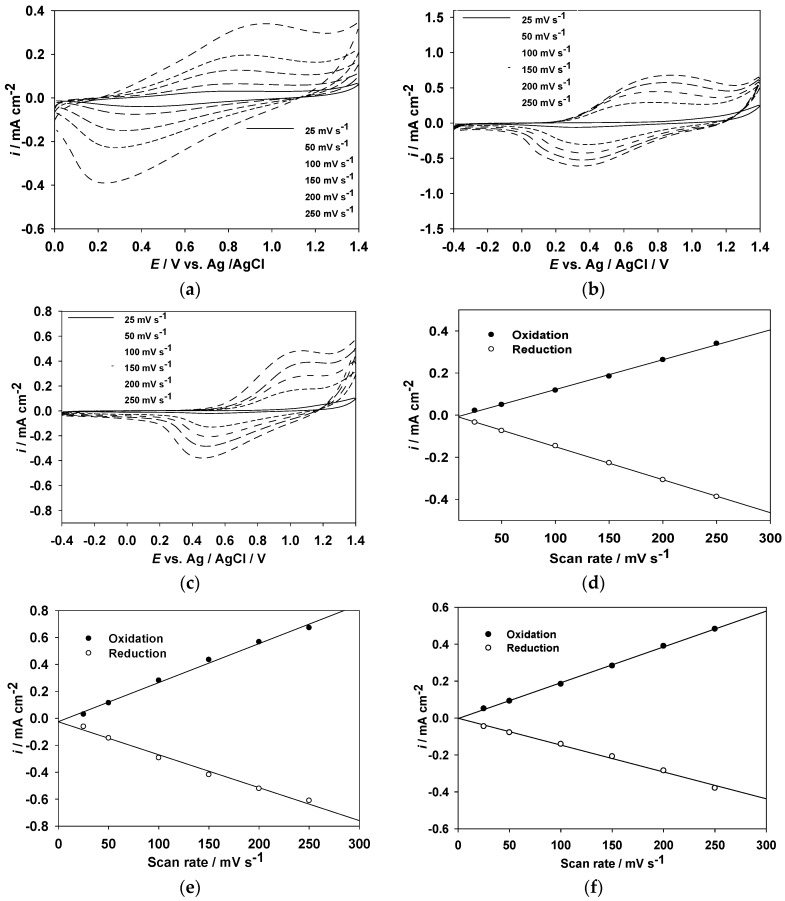
CV curves of (**a**) PMPS; (**b**) PMPO; and (**c**) PANIL films at different scan rates between 25 and 250 mV·s^−1^ in 0.1 M LiClO_4_/EtOH solution, and the relationship between the peak current density and scan rate of (**d**) PMPS; (**e**) PMPO; and (**f**) PANIL films in 0.1 M LiClO_4_/EtOH solution.

Figure 3a–c shows the relationship of the peak current vs. scan rate of PMPS, PMPO, and PANIL films in a 0.1 M LiClO_4_/EtOH solution at scanning rates between 25 and 250 mV·s^−1^. The scan rate dependence of the anodic and cathodic peak current densities shows a linear dependence on the scan rate as depicted in Figure 3d–f, indicating that the redox processes are not diffusion controlled and that the electroactive polymer films are well-adhered on the ITO-coated electrode surface [33].

### 3.2. Electrochromic Properties of PMPS, PMPO, and PANIL Films

**Figure 4:**
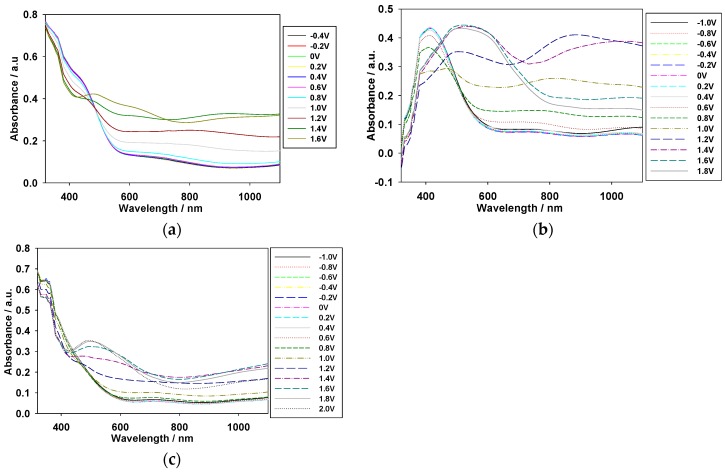
Spectroelectrochemical spectra of (**a**) PMPS; (**b**) PMPO; and (**c**) PANIL films on an ITO electrode at different potentials in [EPI^+^][TFSI^−^] solution.

The absorption spectra of the PMPS, PMPO, and PANIL films coated on an ITO/glass electrode were investigated between −0.4 and +1.6 V in [EPI^+^][TFSI^−^] solution. As shown in Figure 4b, the PMPO film shows an evident π-π* transition peak at around 421 nm. However, the PMPS film shows a shoulder at about 440 nm (Figure 4a); the incorporation of a methylthio group into the polymer backbone causes bathochromic shifts in the absorption band. On the other hand, the incorporation of an electron withdrawing benzonitrile unit into the PSNS backbone deactivates the phenyl unit on the pyrrole ring of PANIL, and the π-π* transition of the PANIL film in [EPI^+^][TFSI^−^] solution shifts hypsochromically to 360 nm.

Upon applying a potential of +0.8 V (vs. Ag/AgCl), the shoulder of the PMPS film at around 440 nm and the absorption peak of the PANIL film at around 360 nm decrease gradually, and charge carrier bands emerge at around 600–1000 nm. Table 1 shows the photos of PMPS, PMPO, and PANIL in the [EPI^+^][TFSI^−^] solution at various potentials. The PMPS film was green (0 V) in the neutral state, dark green (1.2 V) in the oxidation state, and brown (1.6 V) in the highly oxidized state. The PMPO and PANIL films were light green (0 V) in their neutral state, whereas the PMPO and PANIL films were blue (1.6 V) and grey (1.6 V), respectively, in the highly oxidized state. The incorporation of an MPS unit into the PSNS backbone gives rise to darker color than those of the MPO and ANIL units. 

**Figure 5:**
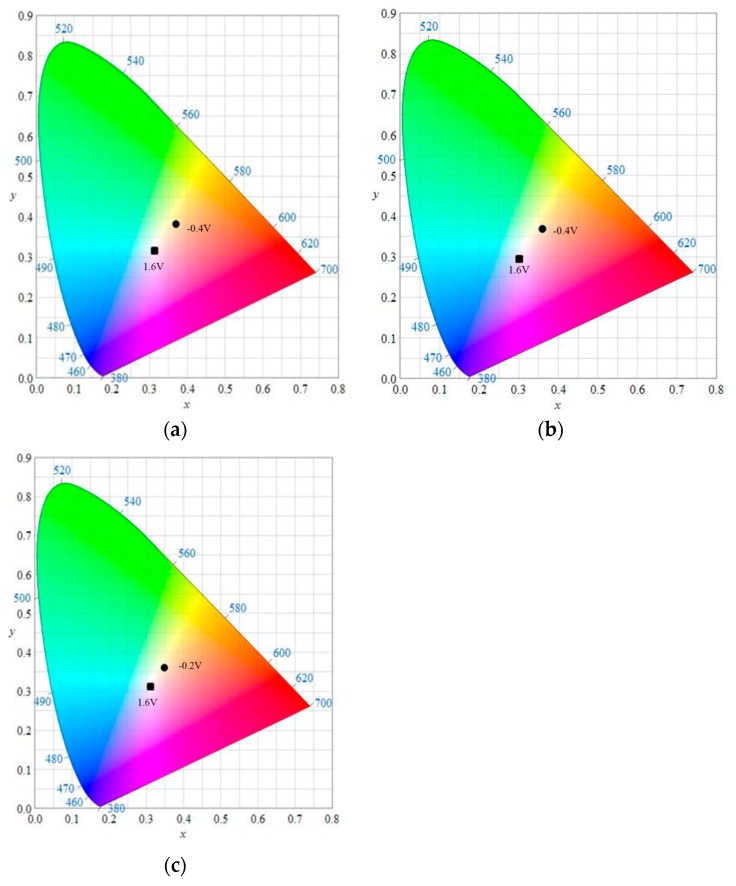
CIE chromaticity diagrams of (**a**) PMPS film in [EPI^+^][TFSI^−^] solution at −0.4 V (●) and 1.6 V (■); (**b**) PMPO film in [EPI^+^][TFSI^−^] solution at −0.4 V (●) and 1.6 V (■); and (**c**) PANIL film in [EPI^+^][TFSI^−^] solution at −0.2 V (●) and 1.6 V (■).

The CIE (Commission Internationale de I'Eclairage) chromaticity diagrams of the PMPS, PMPO, and PANIL films in neutral and oxidation states are shown in Figure 5, and the colorimetric values (*L*, *a*, *b*, L*, a*, and b*) and CIE chromaticity values (*x*, *y*) of the three polymer films at various potentials in the [EPI^+^][TFSI^−^] solution are summarized in Table 2. The b* of the PMPO film was negative between 1.2 and 1.8 V, demonstrating that the PMPO film was blue (1.6 V) in the highly oxidized state.

The optical band gap (*E*_g_) of PMPS, PMPO, and PANIL can be calculated according to the Planck equation [34],

(1) *E*_g_ = 1241/λ_onset_

where λ_onset_ is the wavelength at which the onset of absorption occurs. The *E*_g_ of PMPS, PMPO, and PANIL were 2.25, 2.17, and 2.21 eV, respectively.

The incorporation of methoxyphenyl into the PSNS backbone showed a lower *E*_g_ than those of the methylthio-phenyl and benzonitrile units. The lowest unoccupied molecular orbital (LUMO) and highest occupied molecular orbital (HOMO) energy levels of PMPS, PMPO, and PANIL were determined using cyclic voltammetry. The *E*_HOMO_ was calculated from *E*_onset_ using the formula [35],

(2) *E*_HOMO_ = −e(*E*_onset_ + 4.8 V) (vs. vacuum)

where *E*_onset_ is the onset potential of oxidation. *E*_LUMO_ of the polymers was calculated using the formula,

(3) *E*_LUMO_ = *E*_HOMO_ + *E*_g_

The HOMO energy level of PMPS, PMPO, and PANIL are −4.90, −4.88, and −5.00 eV, respectively, and the LUMO energy level of PMPS, PMPO, and PANIL are −2.65, −2.71, and −2.79 eV, respectively. The PANIL film shows a lower LUMO energy level than those of the PMPS and PMPO films, and this can be attributed to the incorporation of an electron withdrawing cyano group in the ANIL unit that decreases the LUMO energy level significantly.

**Figure 6:**
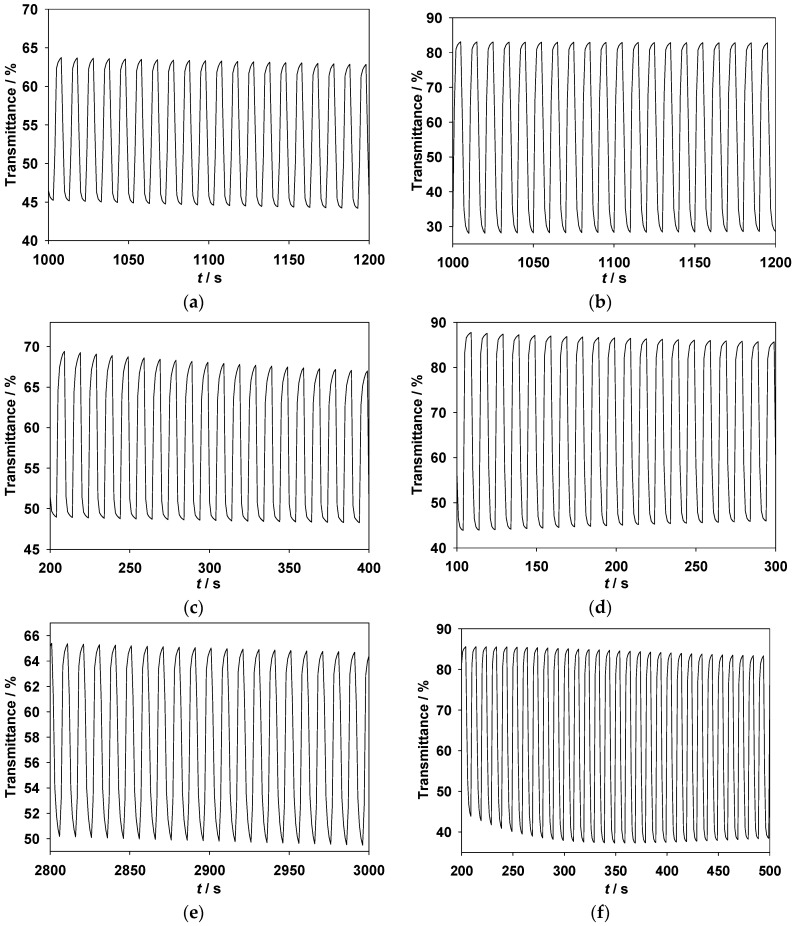
In situ transmittance of (**a**) PMPS film at 600 nm; (**b**) PMPS film at 940 nm; (**c**) PMPO film at 584 nm; (**d**) PMPO film at 950 nm; (**e**) PANIL film at 566 nm; and (**f**) PANIL film at 950 nm as a function of time in [EPI^+^][TFSI^−^] solution. The time interval is 5 s. The conducting polymer films were stepped by repeated potential between −0.2 and +0.9 V.

A square-wave cyclic potential step method accompanied by UV-Vis spectroscopy was used to determine the optical contrast and switching time of the PMPS, PMPO, and PANIL films. The polymer films were immersed in [EPI^+^][TFSI^−^] solution and stepped by repeated potential between neutral and oxidation states with a time interval of 5 s. Figure 6 exhibits the transmittance-time profiles of the PMPS film at 600 and 940 nm, the PMPO film at 584 and 950 nm, and the PANIL film at 566 and 950 nm. The coloration switching time (τ_c_) and bleaching switching time (τ_b_) of the PMPS, PMPO, and PANIL films in the [EPI^+^][TFSI^−^] solution are summarized in Table 3. The optical switching time (*T*_95%_) of the PMPS film is 2.21 and 1.97 s at 600 and 940 nm, respectively, from the bleaching state to the coloring state at the 100th cycle, and 1.93 and 2.01 s at 600 and 940 nm, respectively, from the coloring state to the bleaching state at the 100th cycle.

The optical contrast (Δ*T*%) is an important characteristic in electrochromic applications [36]. The Δ*T*_max_ of the PMPS, PMPO, and PANIL films are 18.62%, 18.02%, and 15.83% at 600 nm, 584 nm, and 566 nm, respectively, in the [EPI^+^][TFSI^−^] solution. Moreover, The Δ*T*_max_ of the PMPS, PMPO, and PANIL films are 54.47%, 43.99%, and 46.17% at 940 nm, 890 nm, and 950 nm, respectively, in the [EPI^+^][TFSI^−^] solution. The PMPS film shows the highest Δ*T*_max_ (54.47% at 940 nm) among these polymer films.

The coloration efficiency (CE) is also a useful parameter in electrochromic applications. CE can be calculated using the following equations at a specific wavelength [37]:

(4) ΔOD = log(*T*_b_/*T*_c_)

(5) η = ΔOD/*Q*_d_

where ΔOD represents the variation of the optical density at a specific wavelength. *T*_b_ and *T*_c_ denote the transmittance of the bleaching state and coloring state, respectively. CE (η) stands for the power efficiency of the electrochromic materials and devices. *Q*_d_ (mC·cm^−^^2^) is the charge density of the electrodes. The η_max_ of the PMPS, PMPO, and PANIL films are 298.28 cm^2^·C^−1^ at 940 nm, 142.48 cm^2^·C^−1^ at 890 nm, and 279.19 cm^2^·C^−1^ at 950 nm, respectively.

### 3.3. Spectroelectrochemistry of ECDs

**Figure 7:**
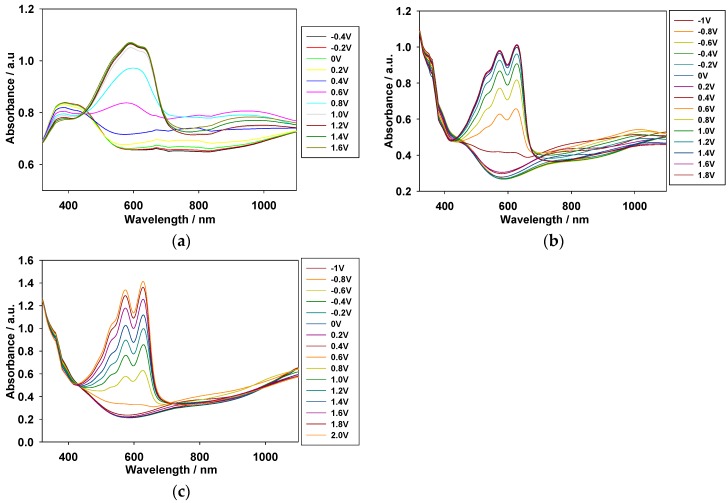
Spectroelectrochemical spectra of (**a**) PMPS/PProDOT-Et_2_; (**b**) PMPO/PProDOT-Et_2_; and (**c**) PANIL/PProDOT-Et_2_ ECDs at various potentials.

**Figure 8:**
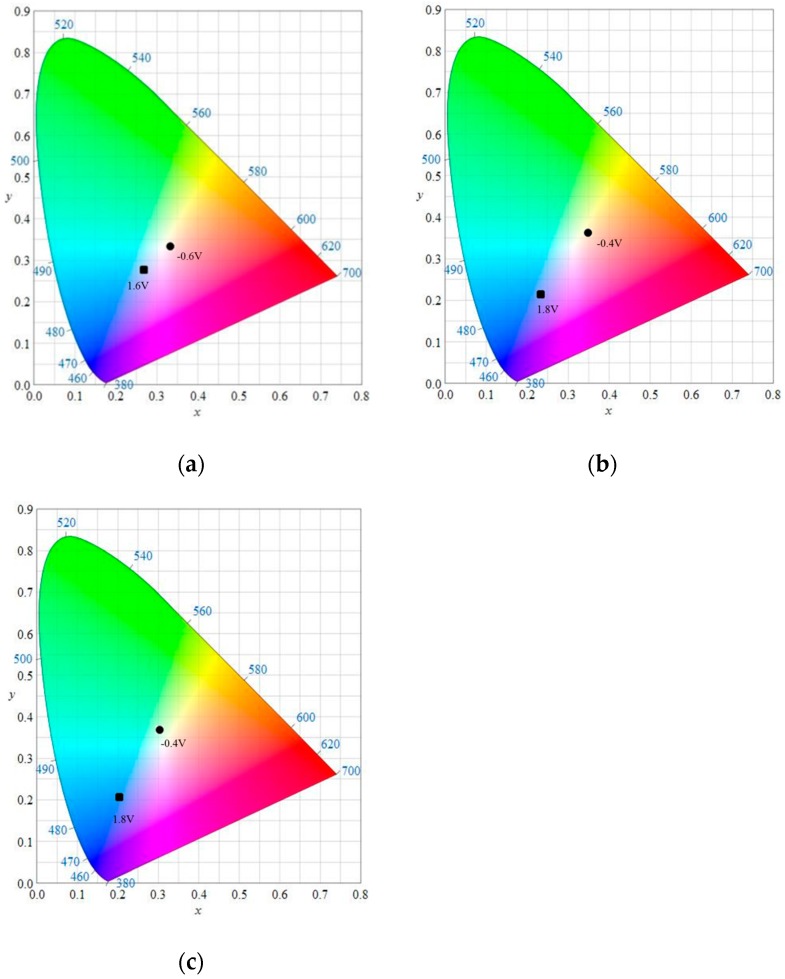
CIE chromaticity diagrams of (**a**) PMPS/PProDOT-Et_2_ at −0.6 V (●) and 1.6 V (■); (**b**) PMPO/PProDOT-Et_2_ at −0.4 V (●) and 1.8 V (■); and (**c**) PANIL/PProDOT-Et_2_ at −0.4 V (●) and 1.8 V (■).

Dual-type ECDs composed of two electrochromic electrodes, one anodically coloring layer (PMPS, PMPO, or PANIL) and the other cathodically coloring material (PProDOT-Et_2_), were facing each other and were separated by an electrolyte membrane. Figure 7a shows the spectroelectrochemical spectra of the PMPS/PProDOT-Et_2_ ECD at potentials between −0.4 V and +1.6 V. PMPS/PProDOT-Et_2_ ECD shows a peak at around 380 nm and a shoulder at around 430 nm at 0 V, and this can be attributed to the π-π* transition peak of the PMPS film in the neutral state. In this situation, PProDOT-Et_2_ was light blue in its oxidation state, and the PMPS/PProDOT-Et_2_ ECD was greyish-green at 0 V. However, the absorption of the π-π* transition peak for the PMPS film lessened and a new peak at 590 nm emerged at +1.6 V, and the PMPS/PProDOT-Et_2_ ECD was cyan at +1.6 V. Under similar conditions, the PMPO/PProDOT-Et_2_ ECD was light green at −0.4 V, bluish-grey at 0.6 V, light blue at 0.8 V, and blue at 1.6 V. The PANIL/PProDOT-Et_2_ ECD was grey at −0.4 V, light blue at 0.8 V, and blue at 1.6 V. The CIE chromaticity values (*x*, *y*) and colorimetric values (*L*, *a*, *b*, L*, a*, b*) of the PMPS/PProDOT-Et_2_, PMPO/PProDOT-Et_2_, and PANIL/PProDOT-Et_2_ dual type ECDs are summarized in Table 4. Moreover, the CIE chromaticity diagrams of the PMPS/PProDOT-Et_2_ ECD at −0.6 and 1.6 V, PMPO/PProDOT-Et_2_ ECD at −0.4 and 1.8 V, and PANIL/PProDOT-Et_2_ ECD at −0.4 and 1.8 V are displayed in Figure 8.

**Figure 9:**
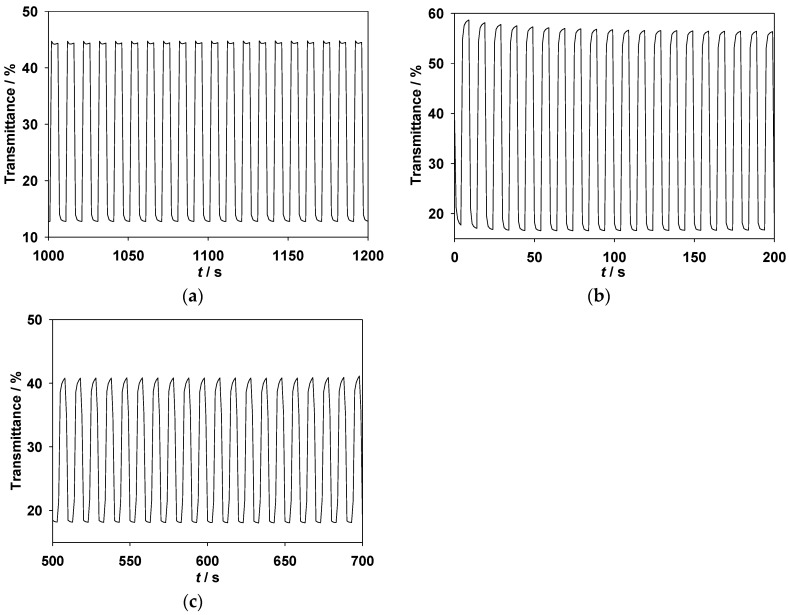
In situ transmittance of (**a**) PMPS/PProDOT-Et_2_ ECD at 590 nm as a function of time, the ECD was stepped by repeated potential between −0.4 and +1.0 V; (**b**) PMPO/PProDOT-Et_2_ ECD at 626 nm, the ECD was stepped by repeated potential between −0.4 and 1.2 V; and (**c**) PANIL/PProDOT-Et_2_ ECDs at 628 nm, the ECD was stepped by repeated potential between 0 and 1.2 V.

The transmittance-time profiles of the PMPS/PProDOT-Et_2_, PMPO/PProDOT-Et_2_, and PANIL/PProDOT-Et_2_ ECDs are shown in Figure 9. The Δ*T*_max_% of the PMPS/PProDOT-Et_2_, PMPO/PProDOT-Et_2_, and PANIL/PProDOT-Et_2_ ECDs were 33% at 590 nm, 41% at 626 nm, and 25% at 628 nm, respectively. The η of the PMPS/PProDOT-Et_2_, PMPO/PProDOT-Et_2_, and PANIL/PProDOT-Et_2_ ECDs, calculated from Equations (1) and (2), were found to be 637.25 cm^2^·C^−1^ at 590 nm, 674.67 cm^2^·C^−1^ at 626 nm, and 401.63 cm^2^·C^−1^ at 628 nm, respectively. The PMPS/PProDOT-Et_2_ and PMPO/PProDOT-Et_2_ ECDs showed higher Δ*T*_max_% and η than those of the PANIL/PProDOT-Et_2_ ECDs, indicating that the incorporations of the methoxyphenyl- and methylthiophenyl-substituted PSNS into the ECDs gave rise to higher Δ*T*_max_% and η than those of the benzonitrile-substituted PSNS. The Δ*T*_max_, ΔOD, and η_max_ of the PMPS/PProDOT-Et_2_, PMPO/PProDOT-Et_2_, and PANIL/PProDOT-Et_2_ ECDs are summarized in Table 5. The τ_c_ and τ_b_ estimated at various double-step potential cycles are listed in Table 3, and the *T*_95%_ of the PMPS/PProDOT-Et_2_ ECD at 590 nm was estimated to be 0.99 s from the bleaching state to the coloring state and 1.01 s from the coloring state to the bleaching state at the 100th cycle. Under similar conditions, the *T*_95%_ of the PMPO/PProDOT-Et_2_ ECD at 626 nm was estimated to be 1.42 s from the bleaching state to the coloring state and 1.12 s from the coloring state to the bleaching state at the 100th cycle, and the *T*_95%_ of the PANIL/PProDOT-Et_2_ ECD at 628 nm was estimated to be 1.17 s from the bleaching state to the coloring state and 1.06 s from the coloring state to the bleaching state. The PMPS/PProDOT-Et_2_ ECD shows shorter τ_c_ than those of the PMPO/PProDOT-Et_2_ and PANIL/PProDOT-Et_2_ ECDs at the 100th cycle, implying that the PMPS/PProDOT-Et_2_ ECD changes color faster from the bleaching state to the coloring state than those of the PMPO/PProDOT-Et_2_ and PANIL/PProDOT-Et_2_ ECDs.

**Figure 10:**
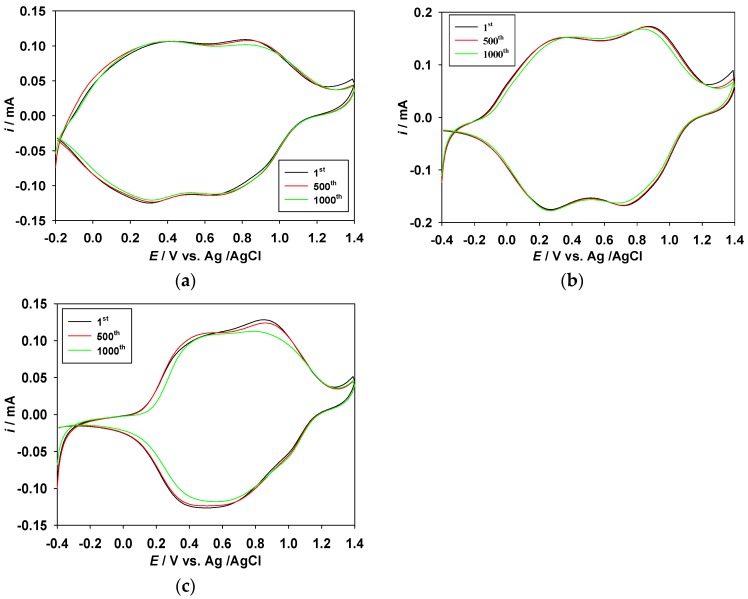
Cyclic voltammograms of (**a**) PMPS/PProDOT-Et_2_; (**b**) PMPO/PProDOT-Et_2_, and (**c**) PANIL/PProDOT-Et_2_ ECDs as a function of repeated scans at 100 mV·s^−1^.

The long-term switching stability of the ECDs between the bleaching and coloring states is an important parameter in practical applications of ECDs [38,39]. The cycling stability of the PMPS/PProDOT-Et_2_, PMPO/PProDOT-Et_2_, and PANIL/PProDOT-Et_2_ ECDs were measured using CV at potentials between −0.4 and +1.4 V with a scan rate of 100 mV·s^−1^. As shown in Figure 10, 94%, 91%, and 90% of the electrical activity was retained after 500 cycles for the PMPS/PProDOT-Et_2_, PMPO/PProDOT-Et_2_, and PANIL/PProDOT-Et_2_ ECDs, respectively, and 91%, 89%, and 87% of the electrical activity was retained after 1000 cycles for the PMPS/PProDOT-Et_2_, PMPO/PProDOT-Et_2_, and PANIL/PProDOT-Et_2_ ECDs, respectively, indicating that the PMPS/PProDOT-Et_2_, PMPO/PProDOT-Et_2_, and PANIL/PProDOT-Et_2_ ECDs displayed reasonable long-term cycling stability. 

**Figure 11:**
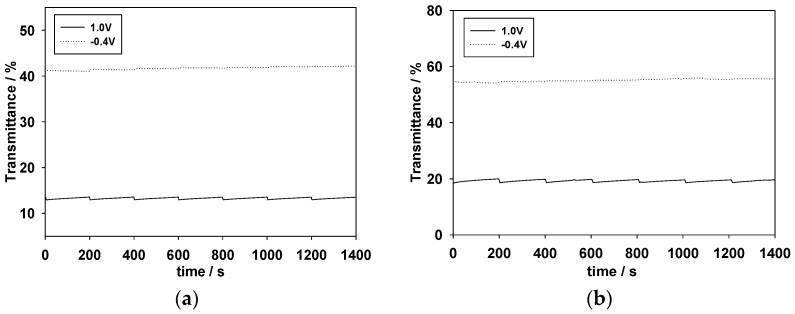

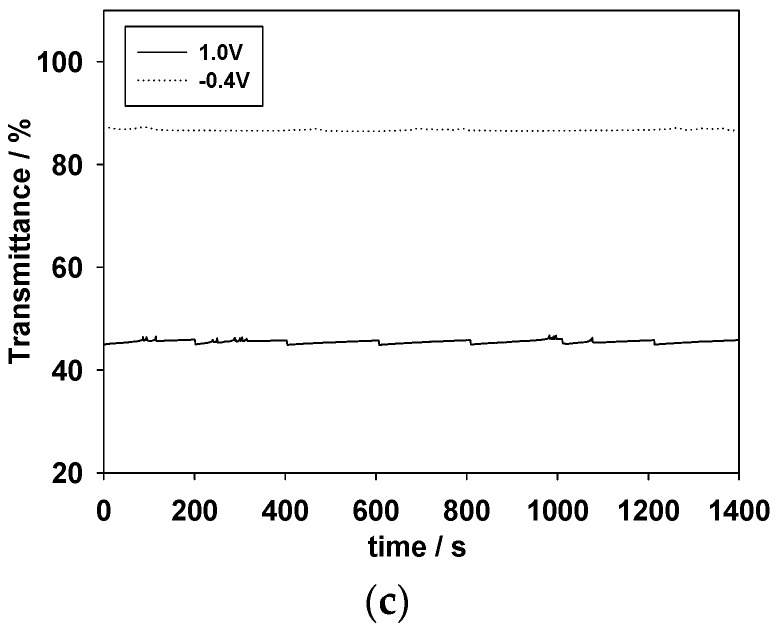
Open circuit stability of (**a**) PMPS/PProDOT-Et_2_ ECD monitored at 590 nm; (**b**) PMPO/PProDOT-Et_2_ ECD monitored at 626 nm; and (**c**) PANIL/PProDOT-Et_2_ ECD monitored at 628 nm.

The optical memory effect is also important for ECD applications [40]. The optical memory of the PMPS/PProDOT-Et_2_, PMPO/PProDOT-Et_2_, and PANIL/PProDOT-Et_2_ ECDs was evaluated at 590, 626, and 628 nm, respectively, with the function of time at −0.4 V and +1.0 V by applying a potential for 1 s for each 200 s time interval. As shown in Figure 11a–c, the PMPS/PProDOT-Et_2_, PMPO/PProDOT-Et_2_, and PANIL/PProDOT-Et_2_ ECDs showed good optical memories in a reduced state of the PMPS, PMPO, and PANIL films, and the transmittance change of the PMPS, PMPO, and PANIL films is less than 1% in their reduced states. However, in the oxidized state of the PMPS, PMPO, and PANIL films and in the reduced state of the PProDOT-Et_2_ film, the PMPS/PProDOT-Et_2_, PMPO/PProDOT-Et_2_, and PANIL/PProDOT-Et_2_ ECDs are less stable than the oxidized state of the PProDOT-Et_2_ film, but the transmittance change is less than 3% in the oxidized state of the PMPS, PMPO, and PANIL films, demonstrating that the PMPS/PProDOT-Et_2_, PMPO/PProDOT-Et_2_, and PANIL/PProDOT-Et_2_ ECDs show reasonable optical memory in the coloring and bleaching states.

## 4. Conclusions

The dithienylpyrrole derivative (MPS) was synthesized via a Knorr-Paal reaction and its corresponding homopolymer (PMPS) was prepared using electrochemical polymerization. According to the spectroelectrochemical analysis, the PMPS, PMPO, and PANIL films revealed distinct electrochromic properties (Δ*T*_max_ ≥ 43.87%) at various potentials in an ionic liquid solution. The PMPS film showed a higher coloration efficiency (η_max_ = 298.28 cm^2^·C^−1^) than those of the PMPO and PANIL films. Dual-type complementary ECDs consisting of PMPS, PMPO, and PANIL films as anodically coloring materials and PProDOT-Et_2_ as the cathodically coloring material were fabricated. Spectroelectrochemical studies showed that the PMPS/PProDOT-Et_2_ ECD is greyish-green and cyan at 0 and +1.6 V, respectively. Electrochromic switching studies showed that the PMPS/PProDOT-Et_2_ ECD exhibited high Δ*T*_max_ (32.51%) and coloration efficiency (637.25 cm^2^·C^−1^) at 590 nm. Considering these results, PMPS film is a promising anodic layer for electrochromic applications.

## Figures and Tables

**Table 1:** Electrochromic behaviors of PMPS, PMPO, and PANIL films in [EPI^+^][TFSI^−^] solution at 0 V and +1.6 V.

Polymer Films and ECDs	Reduction (0 V)	Oxidation (+1.6 V)
PMPS	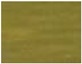	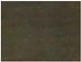
PMPO	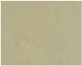	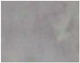
PANIL	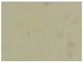	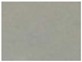
PMPS/PProDOT-Et_2_	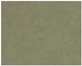	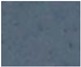
PMPO/PProDOT-Et_2_	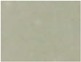	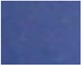
PANIL/PProDOT-Et_2_	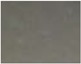	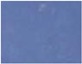

**Table 2:** The colorimetric values (*L*, *a*, *b*, L*, a*, b*) and CIE chromaticity values (*x*, *y*) of PMPS, PMPO, and PANIL films at different applied potentials in [EPI^+^][TFSI^−^].

Polymers	*E*/V	*L*	*a*	*b*	L*	a*	b*	*x*	*y*
PMPS	−0.4	80.25	−1.00	25.76	84.17	0.09	31.17	0.3726	0.3828
	−0.2	80.24	−0.92	25.75	84.17	0.17	31.16	0.3727	0.3827
	0	80.37	−1.16	25.83	84.27	−0.08	31.25	0.3724	0.383
	0.2	80.26	−0.93	25.73	84.18	0.16	31.12	0.3726	0.3826
	0.4	80.32	−1.06	25.72	84.23	0.03	31.1	0.3723	0.3826
	0.6	80.19	−1.35	25.35	84.13	−0.28	30.55	0.3708	0.3819
	0.8	79.38	−1.78	23.81	83.45	−0.73	28.4	0.3663	0.3784
	1.0	76.51	−1.99	18.99	81.03	−1.01	21.93	0.3541	0.3666
	1.2	72.95	−1.61	13.51	78	−0.66	14.98	0.3413	0.3526
	1.4	67.69	−0.39	6.78	73.42	0.57	6.93	0.3269	0.3346
	1.6	64.25	1.41	3.69	70.37	2.52	3.40	0.3227	0.3251
PMPO	−0.4	84.19	0.76	24.26	87.42	1.91	28.09	0.3677	0.3731
	−0.2	84.19	0.74	24.29	87.42	1.9	28.13	0.3678	0.3732
	0	84.16	0.7	24.31	87.4	1.85	28.15	0.3677	0.3733
	0.2	84.16	0.56	24.23	87.4	1.71	28.04	0.3673	0.3733
	0.4	83.98	−0.05	23.49	87.25	1.1	27.01	0.3645	0.3719
	0.6	83.12	−0.97	21.06	86.55	0.15	23.77	0.3571	0.3667
	0.8	81	−1.31	16.05	84.8	−0.22	17.39	0.3447	0.3549
	1.0	75.3	−0.14	5.62	80.01	0.92	5.23	0.3228	0.3296
	1.2	67.81	2.85	−2.19	73.53	4.07	−3.2	0.31	0.3087
	1.4	61.26	3.77	−4.04	67.67	5.12	−5.21	0.3069	0.3024
	1.6	61.06	4.08	−3.24	67.49	5.46	−4.36	0.3095	0.3041
	1.8	61.94	4.08	−2.58	68.29	5.45	−3.64	0.3113	0.306
PANIL	−0.4	87.66	−0.42	20	90.25	0.76	21.69	0.3525	0.3606
	−0.2	87.71	−0.44	20.01	90.29	0.74	21.7	0.3525	0.3606
	0	87.74	−0.47	20.03	90.32	0.71	21.71	0.3525	0.3606
	0.2	87.79	−0.52	20.03	90.35	0.66	21.71	0.3524	0.3606
	0.4	87.78	−0.64	19.96	90.35	0.55	21.62	0.352	0.3606
	0.6	87.69	−0.93	19.58	90.28	0.25	21.13	0.3507	0.3599
	0.8	87.11	−1.29	18.28	89.81	−0.11	19.55	0.3473	0.3573
	1.0	85.34	−1.45	15.21	88.36	−0.3	15.9	0.3406	0.3508
	1.2	80.64	−0.96	8.74	84.5	0.13	8.55	0.3277	0.3365
	1.4	74.34	0.8	3.1	79.19	1.9	2.44	0.3185	0.3229
	1.6	70.62	3.46	1.04	75.99	4.7	0.25	0.3186	0.3161
	1.8	69.85	5.35	1.53	75.32	6.69	0.81	0.3231	0.3158
	2.0	70.35	5.88	2.85	75.76	7.24	2.26	0.3272	0.3185

**Table 3:** Color-bleach kinetics of PMPS, PMPO, and PANIL films in [EPI^+^][TFSI^−^] and ECDs.

Polymer Films and ECDs	λ_max_/nm	Cycle No.	Optical Contrast/%	τ_c_/s	Optical Contrast/%	τ_b_/s
			*ΔT*/%	*T*_95%_	*ΔT*/%	*T*_95%_
PMPS film in [EPI^+^][TFSI^−^]	600	1	17.59	2.22	17.59	2.01
		50	17.27	2.16	17.28	2.08
		100	18.62	2.21	18.61	1.93
	940	1	53.94	1.98	53.94	2.09
		50	53.1	1.96	53.1	2.07
		100	54.47	1.97	54.47	2.01
PMPO film in [EPI^+^][TFSI^−^]	584	1	18.02	2.05	18.02	1.76
		50	18.01	2.04	18.03	1.69
		100	16.98	1.92	16.98	1.76
	890	1	43.99	1.85	43.99	2.02
		50	43.45	1.74	43.45	2.13
		100	43.72	1.87	43.87	2.01
PANIL film in [EPI^+^][TFSI^−^]	566	1	15.83	2.05	15.82	2.05
		50	15.26	2.06	15.25	2.09
		100	15.09	2.01	15.09	2.08
	950	1	46.17	1.97	46.17	2.10
		50	44.63	1.94	44.63	2.28
		100	39.44	2.08	39.44	2.14
PMPS/PProDOT-Et_2_ ECD	590	1	32.51	1.00	32.51	1.10
		50	30.43	0.94	30.43	1.00
		100	31.92	0.99	31.91	1.01
PMPO/PProDOT-Et_2_ ECD	626	1	41.13	1.54	41.13	1.10
		50	39.43	1.45	39.43	0.98
		100	38.50	1.42	38.50	1.12
PANIL/PProDOT-Et_2_ ECD	628	1	25.00	1.21	25.00	1.06
		50	22.23	1.14	22.23	1.03
		100	21.71	1.17	21.71	1.06

**Table 4:** The colorimetric values (*L*, *a*, *b*, L*, a*, b*) and CIE chromaticity values (*x*, *y*) of PMPS/PProDOT-Et_2_, PMPO/PProDOT-Et_2_, and PANIL/PProDOT-Et_2_ ECDs at different applied potentials.

ECDs	*E*/V	*L*	*a*	*b*	L*	a*	b*	*x*	*y*
PMPS/PProDOT-Et_2_	−0.6	78.12	−1.07	11.74	82.39	−0.02	12.28	0.3354	0.3449
	−0.4	78.13	−1.13	11.79	82.4	−0.08	12.33	0.3355	0.345
	−0.2	78.13	−1.27	11.77	82.4	−0.23	12.31	0.3352	0.3451
	0	78	−1.67	11.35	82.3	−0.65	11.8	0.3335	0.3444
	0.2	77.44	−2.18	10.21	81.82	−1.19	10.48	0.33	0.3421
	0.4	75.55	−1.87	7.83	80.22	−0.9	7.77	0.3252	0.3364
	0.6	69.94	0.1	1.09	75.4	1.13	0.32	0.3129	0.3189
	0.8	64.41	−0.34	−6.29	70.52	0.58	−7.49	0.2942	0.3009
	1.0	61.4	0.1	−10.71	67.8	1.05	−11.97	0.2839	0.2893
	1.2	60.73	0.72	−12.22	67.18	1.73	−13.46	0.2814	0.2851
	1.4	60.42	1.34	−13.27	66.91	2.43	−14.47	0.2801	0.2822
	1.6	60.06	1.91	−13.56	66.57	3.07	−14.76	0.2804	0.2809
PMPO/PProDOT-Et_2_	−0.4	69.99	−1.97	16.09	75.44	−1.11	18.83	0.3498	0.3626
	−0.2	69.92	−2.32	15.75	75.38	−1.49	18.37	0.3482	0.362
	0	69.45	−2.75	14.84	74.97	−1.97	17.17	0.3449	0.3599
	0.2	67.75	−2.44	12.62	73.48	−1.68	14.35	0.3397	0.3538
	0.4	61.31	−1.16	5.04	67.72	−0.38	5.12	0.322	0.332
	0.6	51.91	−1.54	−7.26	58.93	−1	−8.84	0.2851	0.2958
	0.8	46.45	−0.32	−14.88	53.58	0.38	−16.8	0.2641	0.2704
	1.0	42.8	1.68	−19.68	49.88	2.84	−21.55	0.2532	0.2526
	1.2	40.43	3.4	−22.6	47.43	5.03	−24.36	0.2476	0.2409
	1.4	38.94	4.52	−24.8	45.86	6.47	−26.39	0.2431	0.2326
	1.6	38.48	4.79	−25.17	45.37	6.82	−26.75	0.2423	0.2307
	1.8	38.23	5.13	−25.7	45.1	7.26	−27.22	0.2415	0.2288
PANIL/PProDOT-Et_2_	−0.4	74.98	−4.17	19.46	79.74	−3.36	22.84	0.3526	0.3715
	−0.2	75	−4.33	19.37	79.75	−3.53	22.71	0.352	0.3713
	0	74.85	−4.6	19.02	79.63	−3.83	22.24	0.3506	0.3706
	0.2	74.5	−4.9	18.3	79.33	−4.16	21.27	0.3482	0.369
	0.4	73.33	−4.61	17.25	78.33	−3.89	19.97	0.3463	0.3664
	0.6	66.93	−2.76	11.09	72.76	−2.05	12.41	0.3349	0.3498
	0.8	55.45	−4.98	−2.59	62.29	−5.02	−3.69	0.2916	0.3135
	1.0	48.59	−4.69	−11.81	55.7	−5.01	−13.66	0.2634	0.2843
	1.2	44.1	−2.96	−18.32	51.2	−3.01	−20.21	0.2465	0.2615
	1.4	40.04	−0.32	−24.33	47.01	0.28	−25.89	0.2334	0.2392
	1.6	35.72	3.14	−30.97	42.4	4.81	−31.75	0.2204	0.214
	1.8	32.91	5.48	−35.32	39.29	7.97	−35.34	0.212	0.1975

**Table 5:** Comparisons of the Δ*T*_max_ and η_max_ for various polymer films and ECDs.

Polymer Films and ECDs	λ/nm	*E*_g_/eV	Δ*T*_max_/%	ΔOD_max_/%	η_max_/cm^2^·C^−1^
PMPS	940	2.17	54.74	46.40	298.28
PMPO	890	2.25	43.87	30.04	142.48
PANIL	950	2.21	44.63	34.94	279.19
PMPS/PProDOT-Et_2_ ECD	590	-	32.51	54.45	637.25
PMPO/PProDOT-Et_2_ ECD	626	-	41.13	52.40	674.67
PANIL/PProDOT-Et_2_ ECD	628	-	25.00	29.80	401.63
